# Effects of Temperature and Applied Potential on the Stress Corrosion Cracking of X80 Steel in a Xinzhou Simulated Soil Solution

**DOI:** 10.3390/ma15072560

**Published:** 2022-03-31

**Authors:** Yuan Cheng, Peng Liu, Mengmeng Yang

**Affiliations:** National Center for Materials Service Safety, University of Science and Technology Beijing, Beijing 100083, China; pengliu@ustb.edu.cn (P.L.); mengmengyang@ustb.edu.cn (M.Y.)

**Keywords:** stress corrosion cracking (SCC), pipeline steel, soil environment

## Abstract

In this research, the stress corrosion cracking (SCC) behavior of X80 pipeline steel in a Xinzhou soil environment at different temperatures and applied potentials was studied with a slow strain rate test (SSRT), potentiodynamic polarization curve measurements, and scanning electron microscopy (SEM). When a higher anodic potential was applied, anodic dissolution occurred at the crack tip and on the crack wall. The cracking mechanism of X80 steel in Xinzhou soil solution is anodic dissolution (AD). At positive cathodic potentials, X80 steel is under an anodic polarization state at the crack tip and under a cathodic polarization state at the crack wall. The SCC of X80 steel is affected by the combined effects of anodic dissolution (AD) and hydrogen embrittlement (HE). At more negative cathodic potentials, both crack tips and crack walls are under cathodic polarization. The SCC of X80 steel is dominated by hydrogen embrittlement (HE). SCC susceptibility has the same variation trend with potentials at different temperatures. The susceptibility to SCC increases notably as the temperature increases at weak cathodic potentials and open circuit potential due to the effect of temperature on the corrosion potential and the diffusion of atoms.

## 1. Introduction

Stress corrosion cracking (SCC) is a type of localized corrosion that has been recognized as one of the most serious threats to the safety of pipelines [1,2,3,4]. At present, it is known that SCC is the result of the interaction of tensile stress with a corrosive environment on a susceptible metallic surface, which results in the initiation and propagation of cracks [5]. In this situation, the pipes will come into contact with soil electrolytes due to tape coating disbondment [4], and then the formation and propagation of SCC will take place.

There are two kinds of SCC: high-pH SCC, which occurs in concentrated carbonate electrolytes, and near-neutral-pH SCC, which occurs in diluted electrolytes [4,6,7,8,9,10]. Intergranular cracking and transgranular cracking can happen in the high-pH SCC and near-neutral-pH SCC processes, respectively [4].

The high-pH SCC mechanism has been identified as anodic dissolution, a conclusion that is widely accepted [11,12,13,14,15,16,17]. A primary model of high-pH SCC has been proposed and revised by various researchers [6,18,19]. During the propagation process, the stability of the passive film formed at crack tips and in other regions differs. Crack growth can be dominated by repeated film rupturing occurring at the crack tip [13,20]. Arafin and Szpunar [17] suggested that the grain boundary plays a key role in the intergranular stress corrosion cracking of pipeline steel. Song et al. and Zhang et al. [11,14] also proposed that stress may enhance the anodic dissolution that takes place at the crack tip as an anode and the region ahead of the crack as a cathode.

Most studies [21,22,23,24,25,26,27,28,29,30,31,32,33,34,35,36] have been carried out on SCC in NS4 solutions. However, there is still no well-accepted mechanism for near-neutral-pH SCC. Most researchers agree that the SCC that takes place in near-neutral-pH environments is dominated by the mixed effects of anodic dissolution (AD) and hydrogen embrittlement (HE) and is due to the synergistic effects of stress, hydrogen, and the local dissolution of the metal [21,22,29,30,31,34,35,36]. Tang et al. [21,22] studied the effects of hydrogen and stress on local dissolution and quantified the contributions of hydrogen, stress, and their synergism to the dissolution rate of steel at the crack tip. Liu et al. [34] studied the SCC mechanism of different pipeline steels and demonstrated that the difference between crack tips and other regions in terms of electrochemical statuses leads to the combined effect of hydrogen and dissolution. Parkins et al. [35] suggested that, in dilute solutions, pH dissolution and hydrogen are involved in the crack growth mechanism. Hydrogen plays a significant role in the cracking of pipeline steel. Mao and Li [36] proposed that hydrogen plays an important role in the process of SCC for pipeline steel by promoting stress corrosion cracking and anodic dissolution. However, some researchers still believe that the local anodic dissolution of the metal has more effect on crack growth in weakly acidic and neutral electrolytes [32,33]. Bogdanov et al. [32] proposed that the crack growth that occurs in a weakly acidic electrolyte is determined by the metal dissolution process. Hydrogen indirectly affects crack growth [32]. Ryakhovskikh et al. [33] pointed out that crack initiation occurs at sites of local corrosion defects. The local dissolution of the metal is the primary crack growth mechanism in X70 pipeline steel under small amplitudes of loading.

Most previous studies on stress corrosion cracking (SCC) have focused on the use of laboratory simulation solutions [37,38,39,40,41,42,43,44]. It has been suggested that a simulated solution for high-pH SCC in laboratory tests is 1 M NaHCO_3_ + 0.5 M Na_2_CO_3_ [18,23,38,40]. NS4 solution (KCl 0.122 g/L, NaHCO_3_ 0.483 g/L, CaCl_2_·2H_2_O 0.181 g/L, MgSO_4_·7H_2_O 0.131 g/L) is often applied as a simulated solution for laboratory research [24,25,37,39].

There has been some research on the SCC of pipelines using simulated soil solutions based on typical soil in China. Liu et al. and Liang et al. [43,45,46,47,48,49,50] studied the SCC mechanism of pipeline steels in acidic and alkaline environments, respectively, based on the soil environment of China. They proved that high-strength pipeline steels were highly susceptible to SCC in the typical soil environments of China. The SCC mechanism in acidic environments was dependent on the potential. Anodic dissolution (AD), hydrogen embrittlement (HE), and the mixed effect of AD and HE all have different effects on SCC at different potentials [47,48,49,50]. Liang et al. [43] proposed that the SCC occurring in alkaline environments is different from traditional high-pH SCC. Additionally, pitting was found to be an important factor in the initiation of cracks.

The factors influencing the SCC of pipelines are complex and include the microstructure, pH value, temperature, and potential. The factors listed above have been studied by many researchers [50,51,52,53,54,55,56,57,58,59]. The influence of microstructure on SCC is mainly caused by oxidation and inclusion, which can result in the initiation of cracks in which hydrogen is prone to becoming trapped. Meanwhile, microstructures with coarse grains and high dislocation densities are more susceptible to SCC [50,51,52]. A hydrogen evolution reaction will be promoted at lower pH values [49]. Many laboratory studies and fieldwork surveys have shown that temperature has a great effect on high-pH SCC [57]. However, few studies have focused on the effect of temperature on SCC. Cathodic protection, which is an effective method with which to protect a pipe, has been widely used on buried pipelines. However, an unsuitable cathodic protection potential can be harmful to a pipeline and promote the occurrence of SCC [58]. Several studies have focused on the effect of potential on SCC [39,53,54,55,56] and proposed suitable cathodic potentials for restricting SCC [53,54,55,56]. The most popular opinion at present is that a strong cathodic polarization potential will increase the susceptibility of pipeline steel to SCC as well as the hydrogen embrittlement risk.

As mentioned above, a large number of studies have been carried out on this topic. However, the environments used are mainly based on a standard solution. Additionally, few environments used in the studies have matched the typical corrosion situation in China; both of these environments are different from the environments in which pipes are buried.

The West-East Gas Pipeline runs through different natural regions and is thousands of kilometers in length. It passes through a number of different provinces in China where the soil environment is very complex. Therefore, it is important to study the SCC of pipeline steel in the soil environment of the regions in which this pipeline is buried.

Xinzhou, in Hubei province, lies in central China. The yellow-brown soil of Xinzhou has a weak acidity and a near-neutral pH of 5.98. However, few studies have focused on the SCC behavior of X80 steel in a weakly acidic soil environment in central China. The pipeline running through this region is long. For the safety of the pipeline, it is important to know the SCC caused by the soil environment.

This paper aims to discuss the SCC behavior of a pipeline under the different applied cathodic potentials and different temperatures of the soil environment of Xinzhou, where this pipe is buried. These results should help us to improve the safety of the pipeline and make better choices about appropriate protection methods.

## 2. Materials and Methods

### 2.1. X80 Pipeline Steel

**Figure 1:**
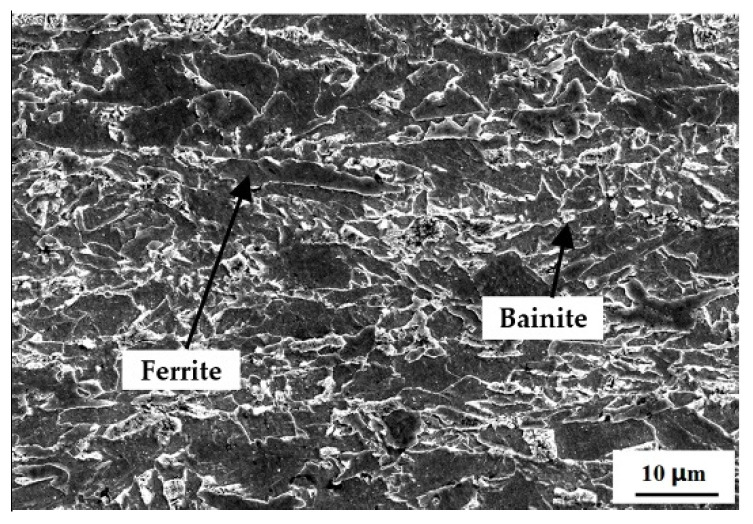
Microstructure of X80 pipeline steel.

The testing specimens were cut from a longitudinally welded X80 pipe (Φ1219 × 22 mm) used in the West-East Gas Pipeline Project II, with the flowing chemical composition (wt.%) C 0.076, Si 0.21, Mn 1.65, S 0.0024, P 0.011, V 0.044, Ti 0.013, Nb 0.048, Ni 0.24, Cu 0.20, Mo 0.22, Cr 0.13, B 0.0003, Al 0.022, N 0.0058, and Fe balance. At room temperature, some of the main mechanical properties of the steel were obtained as follows: ultimate tensile strength, 754 MPa; yield strength, 694 MPa; elongation, 17.8%; and reduction in area, 71.3%. The microstructure of the X80 steel contained an acicular ferrite and a small amount of bainite, as shown in Figure 1.

### 2.2. Soil Environment

Soil samples were collected from the Xinzhou region where the pipelines of West-East Pipeline Project II were buried at a depth of 2 m underground. The chemical composition of the simulated Xinzhou soil solution was (g/L) NaHCO_3_, 0.0716; MgSO_4_·7H_2_O, 0.0769; CaCl_2_, 0.0165. The solution was made from analytic-grade reagents and ultra-pure water. The pH of the solution was adjusted to 5.98 ± 0.10 using HAC or 5% sodium hydroxide (NaOH).

### 2.3. Electrochemical Test

Electrochemical tests were conducted using CHI 660D (CHI, Shanghai, China) in an electrochemical three-electrode cell which consisted of an X80 steel working electrode (WE) with a working area of 1.0 cm^2^, a platinum (Pt) plate counter electrode (CE), and a saturated calomel electrode (SCE) reference electrode (RE). All the potentials in this paper refer to SCE. The specimens used in the electrochemical tests were polished sequentially with 800 grit emery papers, followed by cleaning with distilled water and absolute ethyl alcohol. Polarization curves were obtained in simulated soil solution at two different scanning rates, 50 mV/s and 0.5 mV/s, which represented fast and slow sweep rates, respectively. The electrochemical tests were performed at different temperatures, which were controlled by a water bath.

### 2.4. Slow Strain Rate Test (SSRT)

**Figure 2:**
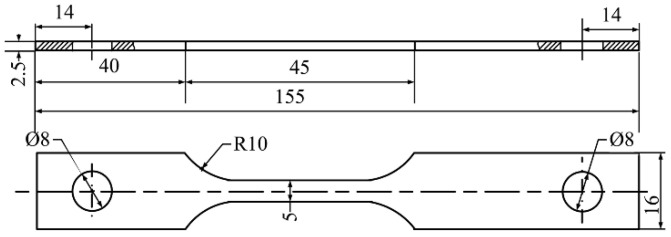
The dimensions of the specimen (mm).

The dimensions of the specimen used for the SSRT are shown in Figure 2. Prior to testing, the gauge lengths of the specimens were polished sequentially with 800 grit emery papers along the tensile direction on all faces, then cleaned using absolute ethyl alcohol and distilled water before finally being dried in air.

The tests were conducted using the WDML-3 (Letry, Xi’an, China) test machine with a maximum load of 30 kN. Before the SSRT experiment, the specimen was loaded with a preload of 300 N to eliminate the gap between the machine and fixture. The SSRTs were performed at 10 °C, 25 °C, and 40 °C, respectively, in Xinzhou simulated soil solution under different potentials, which were −400 mV, −550 mV, −850 mV, −1000 mV, and −1150 mV. Additionally, in order to study the material’s susceptibility to SCC, the SSRT was also performed without an applied potential, representing the open circuit potential (OCP), and in air. The strain rate used in both air and solution was 7.5 × 10^−7^ s^−1^. The temperatures in the test were controlled by a water bath cell. The susceptibility of the steel to SCC was evaluated by the ratio of reduction in area, I_φ_, which can be calculated using Equation (1):

(1) $$I_{\varphi }=\left[1-\left(\frac{\varphi_{a}}{\varphi_{0}}\right)\right]\times 100\%$$

where φ_0_ is the reduction in the area of the tensile specimen in air and φ_a_ is the reduction in the area of the tensile specimen in solution. Therefore, susceptibility to SCC increases with I_φ_.

After the SSRTs, the fracture surfaces of the samples subjected to the different conditions were cleaned by an ultrasonic cleaner, and then the fracture and side surfaces of the samples were observed using scanning electron microscopy (SEM) (LEO-1450, German).

## 3. Results

### 3.1. Potentiodynamic Polarization

**Figure 3:**
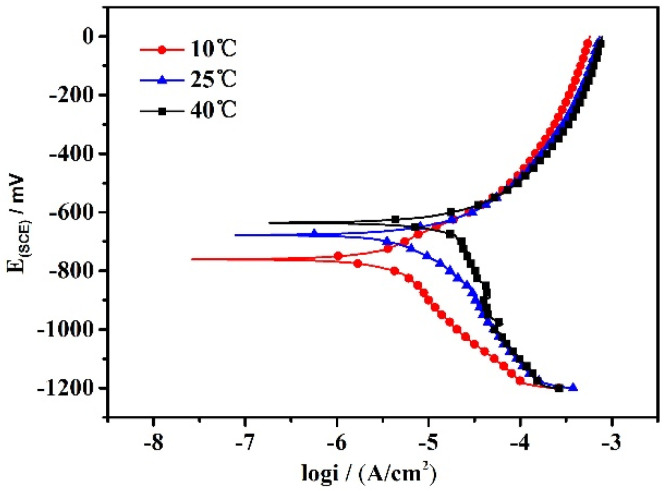
Polarization curves of X80 pipeline steel at different temperatures in the simulated soil solution.

Figure 3 shows the polarization curves of X80 steel at different temperatures in a Xinzhou simulated soil solution. From the graph, it can be seen that the curves had similar features for the X80 steel, in that no protective film formed on the surface of X80 steel in the test solution. The graph shows an active anodic dissolution status at different temperatures.

The electrochemical parameters are listed in Table 1. Both E_corr_ and I_corr_ increased along with the temperature. In particular, I_corr_ increased from 0.502 × 10^−6^ A·cm^−2^ to 4.046 × 10^−5^ A·cm^−2^ as the temperature rose from 10 °C to 40 °C. I_corr_ was proportional to the rate of corrosion. An increase in temperature enhances the activity of corrosive ions such as SO_4_^2−^ and Cl^−^. As a result, the corrosion rate increases obviously with the current density. From Table 1, it can also be seen that the corrosion rate in the simulated soil solution at a temperature of 40 °C is eight times higher than that at 10 °C.

### 3.2. SSRT of X80 Steel Tested at Different Potentials in Simulated Xinzhou Soil Solution

**Figure 4:**
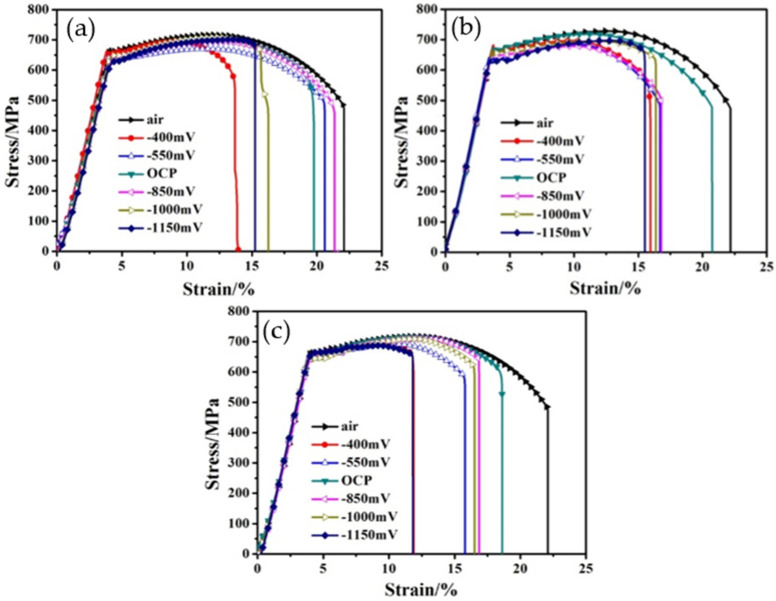
The stress-strain curves of X80 steel with different applied potentials and temperatures: (**a**) 10 °C, (**b**) 25 °C, and (**c**) 40 °C.

Figure 4a shows the stress-strain curves of X80 steel in Xinzhou simulated soil solution at 25 °C under various polarizing potentials and with a strain rate of 7.5 × 10^−7^ s^−1^. Both the strain and strength of the X80 steel decreased compared to those of the steel kept in the air. When the specimens were tested in air, a larger strain was found; this strain was larger than that obtained in the simulated solution at different applied potentials. This shows that X80 steel is susceptible to SCC in a Xinzhou soil solution. The results obtained at 10 °C and 40 °C are similar to the result obtained at 25 °C, as shown in Figure 4b,c.

**Figure 5:**
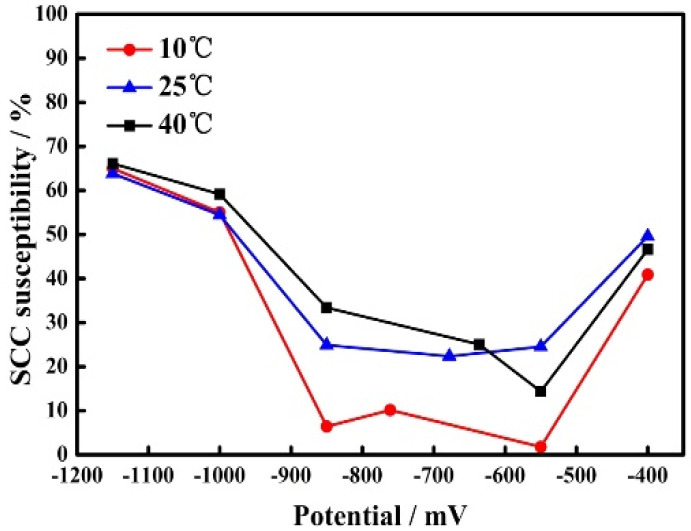
Effects of temperature and applied potential on susceptibility to SCC at 10 °C, 25 °C, and 40 °C.

Furthermore, the effects of temperature and applied potential on susceptibility to SCC in the slow strain rate test carried out at 10 °C, 25 °C, and 40 °C are shown in Figure 5. Apparently, the SCC mechanism of pipelines in the Xinzhou simulated solution depends on the potential applied to the X80 steel. When the potential becomes more negative, the susceptibility to SCC increases. It is noted that between the OCP and −850 mV (which is the common cathodic protection potential used for buried pipelines in engineering), susceptibility to SCC shows no apparent variation. However, when the applied potential decreases from −850 mV to −1150 mV, the susceptibility to SCC increase noticeably, which suggests that SCC is easily induced due to cathodic potential.

### 3.3. SEM Images of X80 Steel after SSRT

**Figure 6:**
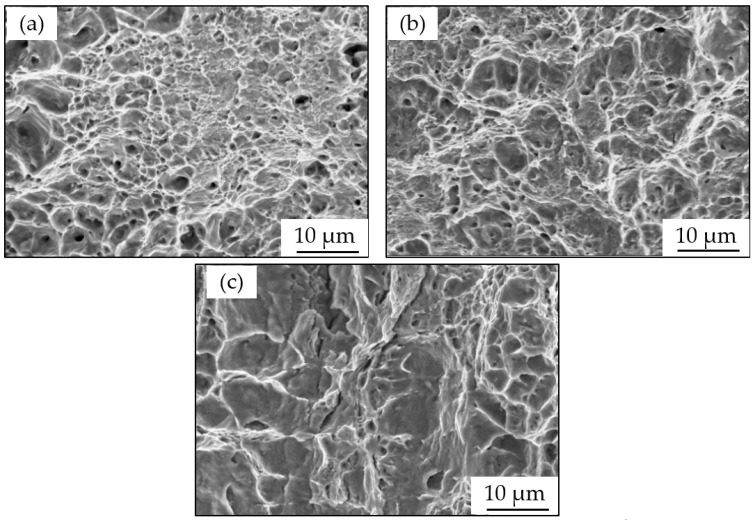
Fracture surface morphology of X80 at different temperatures in a simulated Xinzhou soil solution: (**a**) 10 °C, (**b**) 25 °C, and (**c**) 40 °C.

Figure 6 shows SEM images taken of the surface fracture morphology of X80 at 10 °C, 25 °C, and 40 °C, respectively, after SSRTs were carried out in the Xinzhou simulated soil solution. It can be seen that the fracture surfaces at 10 °C and 25 °C were made up of a great number of small dimples, showing that ductile fractures occurred in Figure 6a,b. However, the dimples shown in Figure 6b, which occurred at 25 °C, are less obvious than the dimples that occurred at 10 °C (as shown in Figure 6a). This demonstrates that the susceptibility to SSC at a temperature of 25 °C is higher than that at 10 °C, as shown in Figure 5. With the rise in temperature, the steel experienced a brittle fracture at 40 °C, as seen in Figure 6c. Based on the analysis above, it can be concluded that the susceptibility of X80 to SCC in simulated Xinzhou soil solution increases when the temperature rises at OCP.

**Figure 7:**
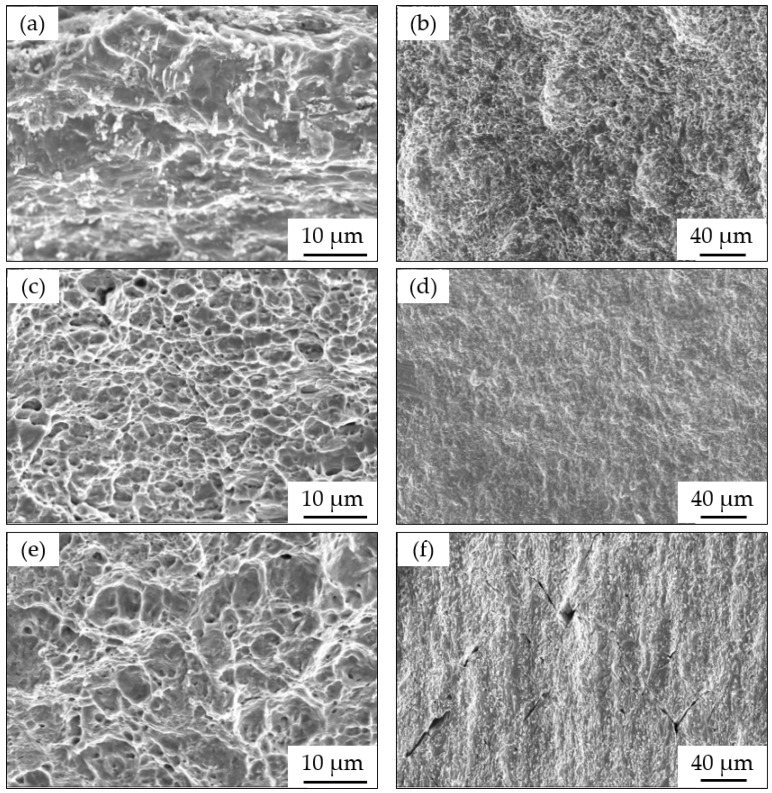
SEM images of X80 with different anode potentials after SSRTs in a simulated Xinzhou soil solution at 25 °C: (**a**) fracture surface at −400 mV; (**b**) side surface at −400 mV; (**c**) fracture surface at −550 mV; (**d**) side surface at −550 mV; (**e**) fracture surface at OCP; and (**f**) side surface at OCP.

Figure 7 shows SEM images of the fracture surfaces and side surfaces of the steel taken after the SSRTs at open circuit potential and different anodic potentials in the simulated Xinzhou soil solution. With the increase in anodic potential applied, the dimples of the fracture surfaces corroded severely. This was especially apparent at the potential of −400 mV, as shown in Figure 7a,b. When a lower anodic potential of −550 mV was applied, dimples appeared on the fracture surface, as shown in Figure 7c. Additionally, the side surface shown in Figure 7d has less corrosion in comparison with the side surface shown at −400 mV. In Figure 7e, dimples on the fracture surface are obvious at OCP. Furthermore, a small amount of secondary cracking appears on the side surface shown in Figure 7f.

**Figure 8:**
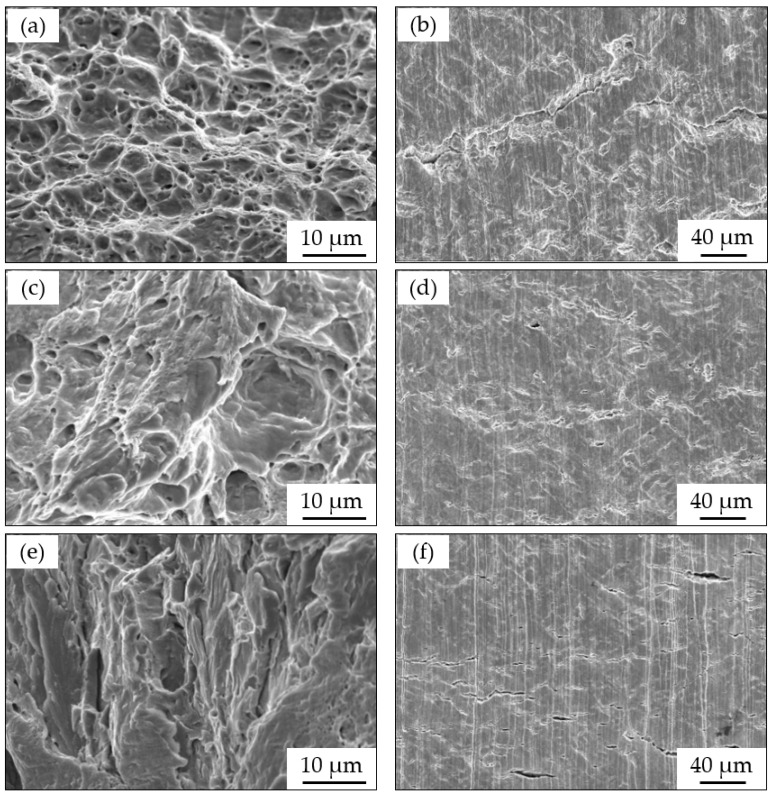
SEM images of X80 with different cathode potentials after SSRTs in a simulated Xinzhou soil solution at 25 °C: (**a**) fracture surface at −850 mV; (**b**) side surface at −850 mV; (**c**) fracture surface at −1000 mV; (**d**) side surface at −1000 mV; (**e**) fracture surface at −1150 mV; and (**f**) side surface at −1150 mV.

Figure 8 shows the SEM images of fracture surfaces and side surfaces taken after the SSRT in a Xinzhou simulated solution with different applied cathodic potentials. At the weak cathode polarization potential of −850 mV, the surface morphology showed a ductile fracture feature with extensive distribution of dimples in Figure 8a. It can be seen that there were only a few cracks in the side surface in Figure 8b. At −1000 mV and −1150 mV, with strong cathodic polarization, the fracture surfaces showed an obvious brittle cracking morphology (see Figure 8c,e). In addition, there were many secondary cracks on the side surfaces perpendicular to the applied stress (see Figure 8d,f).

## 4. Discussion

### 4.1. Effects of Applied Potentials on SCC of X80 Steel in Xinzhou Simulated Solution

**Figure 9:**
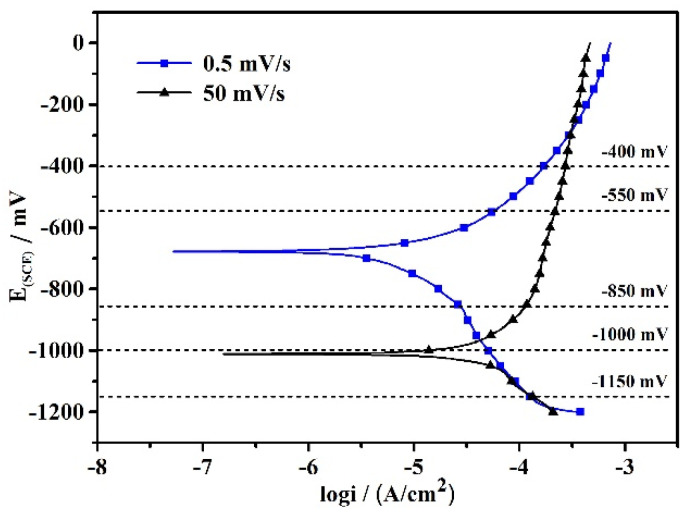
Polarization curves of X80 at fast and slow sweep rates.

A method based on fast and slow scanning rates is generally applied to predict the SCC process of steel. According to Parkins [35], during fast sweeping, there is insufficient time for a film to form on the electrode surface. The steel is in a state of active dissolution which can be used to simulate the crack tip electrochemistry. In contrast, during slow sweeping, film formation is favored and the measured polarization behavior can be used to simulate the crack wall electrochemistry. Liu et al. [10,39,48] also propose that electrochemical tests could be performed at a fast scan rate of 50 mV/s and a slow sweep rate of 0.5 mV/s in order to analyze the SCC mechanism. The fast and slow scanning rate polarization curves obtained can represent the electrochemical features at the crack tip and on the non-cracked tip surface. Therefore, the difference in electrochemistry between the crack tip and crack wall can be judged using the polarization curve at fast and slow sweep rates of 50 mV/s and 0.5 mV/s, respectively (see Figure 9). The corrosion potentials obtained from the fast and slow scanning rate curves were −1005 mV and −687 mV, respectively.

In the Xinzhou simulated soil solution with a low solution pH (5.98) and the presence of bicarbonate ions, the main possible anodic and cathodic reactions occurring on the steel included [10,37,45,60]:

(2) $$\mathrm{Fe}-2e^{-}\to {\mathrm{Fe}}^{2+}$$

(3) $$O_{2}+2H_{2}O+4e^{-}\to 4{\mathrm{OH}}^{-}$$

(4) $$H_{2}O+e^{-}\to H+{\mathrm{OH}}^{-}$$

(5) $${\mathrm{HCO}}_{3}^{-}+e^{-}\to {\mathrm{CO}}_{3}^{2-}+H$$

(6) $${\mathrm{Fe}}^{2+}+{\mathrm{HCO}}_{3}^{-}\to {\mathrm{FeCO}}_{3}+H^{+}$$

(7) $$H^{+}+e^{-}\to H$$

It is apparent that there were no active-passive transitions and that the steel electrode experienced an active dissolution state at both slow and fast sweep rates (see Figure 9). FeCO_3_ formed in solution because of the existence of Fe^2+^ and HCO^−^_3_. However, the FeCO_3_ layers did not form a stable protective film, as in the situation in a near-neutral-pH solution [10,48]. Therefore, there was no passive feature in the polarization curves.

At −400 mV and −550 mV (see Figure 9), the curves for the slow and fast sweep rates were placed in the anodic region which indicated that anodic dissolution occurred at the crack tip and on the crack wall surface. At the same time, the current densities of the two curves were very high. X80 steel has an anodic dissolution mechanism [10,48,60]; as a result, rapid anodic dissolution happened. Due to the anodic dissolution, severe features of corrosion were present on the fracture surface of the SSRT specimens, as shown in Figure 7b,d, where the susceptibility to SCC should be low. The increase in the SCC susceptibility at −400 mV shown in Figure 5 was caused by the anodic dissolution inducing a reduction in dimensions. The calculation result of the reduction in the area increased.

When the applied potentials decreased to −850 mV and −1000 mV (see Figure 9), the anodic dissolution occurred at the crack tip. Meanwhile, the X80 steel was subjected to cathodic polarization from the slow sweep curve at the crack wall. A combined electrochemical process of anodic reaction at the crack tip and cathodic reaction at the crack wall occurred [48]. However, the anodic and cathodic processes had different effects on SCC at different potentials [10,60]. At −850 mV, the higher current density obtained from the fast scan rate curve indicates that anodic dissolution at the crack tip was the major factor in the SCC process. Anodic dissolution at the crack tip accelerated the cracking process. At −1000 mV, a higher current density from the slow scan rate curve indicated that the cathodic reaction was rapid, and the effect of hydrogen was the major reason for SCC. At the same time, the anodic dissolution occurring at the crack tip with a lower current density promoted the SCC process.

With the further negative shift in the potential to −1150 mV (see Figure 9), the steel was subjected to cathodic polarization at both the crack tip and crack wall from the fast and slow sweep curves. Therefore, the cathodic reaction dominated at both the crack tip and crack wall [48]. The hydrogen evolution potential can be calculated as −595 mV [10,11]. Therefore, hydrogen evolution happened at both the crack tip and crack wall. Hydrogen atoms are generated at both the crack tip and crack wall. The steel SCC was dominated by the hydrogen-based mechanism. From the SEM images shown in Figure 8, the fracture surface was characterized by brittle features. Thus, hydrogen was actively involved in the SCC process, typically causing a transgranular SCC feature.

### 4.2. Effects of Temperature on the SCC of X80 Steel in Xinzhou Simulated Solution

The susceptibility to SCC apparently changed from −550 mV to −1000 mV, which is around the OCP. The temperature mainly affected the diffusion of ions, oxygen solubility, and the anodic and cathodic reaction in the solution.

Based on the analyses above, it is clear that the SCC of X80 steel follows an anodic dissolution mechanism at anodic potential [10,48,60]. On the one hand, the temperature promotes solution convection and enhances the diffusion of ions in the Xinzhou simulated solution; the anodic and cathodic reactions will then be promoted. On the other hand, oxygen solubility declines with the rise in temperature which weakens the effect of temperature on cathodic reaction. Therefore, the temperature has unobvious effects on SCC susceptibility at higher anodic polarization (see Figure 5). The abnormal increase in SCC susceptibility caused by the severe anodic dissolution leads to the reduction in dimensions as mentioned above.

The susceptibility to SCC increases apparently with the temperature from −550 mV to −1000 mV, which are around the OCP (see Figure 5). According to the analysis above, at OCP and weak cathodic potentials, SCC was controlled by the combined effects of anodic dissolution at the crack tip and cathodic reactions at the crack wall. The anodic and cathodic processes had different effects on SCC at different potentials [10,48,60]. At −850 mV, anodic dissolution at the crack tip was the major factor in the SCC process. At this potential, the increase in temperature accelerates the anodic dissolution by enhancing the diffusion of ions. The cathodic reaction is also promoted with temperature increases. Therefore, the difference in SCC susceptibility at different temperatures is huge (see Figure 5). At −1000 mV, a more negative potential than the hydrogen evolution potential, the cathodic reaction was rapid with a high cathodic current density (see Figure 9). Hydrogen was the major reason for the SCC process. Meanwhile, the anodic dissolution was weak with a lower current density (see Figure 9). So, the temperature has less effect on anodic dissolution compared with the condition at −850 mV. As a result, there are fewer differences in SCC susceptibility at different temperatures at −1000 mV (see Figure 5).

With the further negative shift of potential to −1150 mV, more hydrogen was generated and affected the SCC process. The anodic dissolution was restricted by cathodic polarization; the temperature had little effect on anodic dissolution. The effects of temperature on SCC were unapparent at different temperatures. Therefore, the susceptibility to SCC showed no apparent differences at different temperatures (see Figure 5).

## 5. Conclusions

1. The SCC mechanism of X80 steel in a Xinzhou simulated soil solution is affected by both anodic dissolution and hydrogen embrittlement.
2. When the potential is higher than −550 mV, the applied potential is anode potential for the crack tip and crack wall and the susceptibility to SCC is low.
3. When the potential is between −850 mV and −1000 mV, the SCC process is promoted by a combined effect of anodic reaction at the crack tip and cathodic reaction at the crack wall. The anodic and cathodic processes have different effects on SCC at different potentials.
4. At the more negative potential of −1150 mV, which is a cathodic polarization for both the crack tip and the crack wall, the SCC of X80 steel is mainly affected by hydrogen embrittlement.
5. The temperature has an unapparent effect on the variation in SCC susceptibility with the applied potentials at different temperatures. However, there is an apparent change in the susceptibility to SCC, which increases as the temperature rises around the weak cathodic polarization area and at the open circuit potential.

## Figures and Tables

**Table 1:** Electrochemical parameters at different temperatures.

T/°C	E_corr_/mV	I_corr_/(A·cm^−2^)
10	−761	0.502 × 10^−6^
25	−678	1.603 × 10^−5^
40	−636	4.046 × 10^−5^

## Data Availability

Not applicable.
